# Commentator Discussion: Real-world localization of cancer in the lung with a commercially available folate receptor-targeted fluorescent agent for intraoperative molecular imaging

**DOI:** 10.1016/j.xjtc.2025.03.003

**Published:** 2025-03-13

See Article page 161.

Presenter: Dr Nicholas Baker

**Unidentified Speaker 1**. This study and manuscript will be discussed by Dr Bryan Burt from UCLA.

**Dr Bryan Burt***(Los Angeles, Calif)*. Good evening. That was a great presentation, Nick, and congratulations, and congratulations to Dr Sangala. I think this technology is potentially revolutionary. I mean, just think about ourselves in 20 years. I'll be looking at ourselves now, our current standards for intraoperative nodule localization, and particularly, assessment of intraoperative margins. I mean, I'd even submit that our current process for evaluating margins by frozen section is antiquated. And it's multistep. It's frustrating. It's labor intensive, and it's time consuming. So, my first 3 questions are about intraoperative margin assessment. In the real world, how confident are you in your margin assessment by this technology? And would you, in the case where you would send frozen section, be so confident that you would not have frozen section if you had a nice determined margin from your glow?

**Figure:**
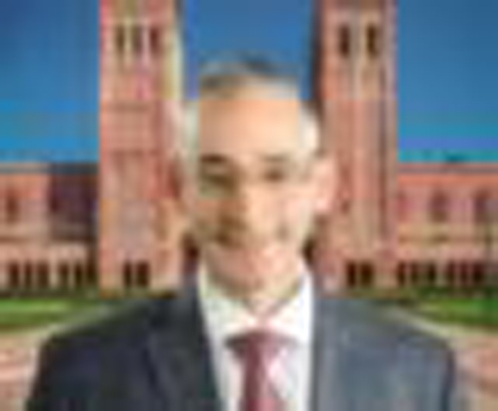

**Dr Nicholas Baker***(Pittsburgh, Pa)*. Yeah. Those are very good questions. And just to give you a little bit of insight to the pathology statement before I answer the questions, I actually brought the pathologists in and showed them this technology, and they were questioning the reps in the room if they could get 1 of these cameras in their frozen suite. But yeah, no, as we progressed, I have gotten more and more lax on sending frozen section on these lesions because we're able to visualize it. And we did have 2 patients who had a close margin, and we took extra at the time, and that was taken before the frozen was back. We knew that it was close as we were stapling it. And we just took a little bit of extra. We always look at these lesions on the back table. We almost always cut into them. Then kind of see where the edge is.

**Figure:**
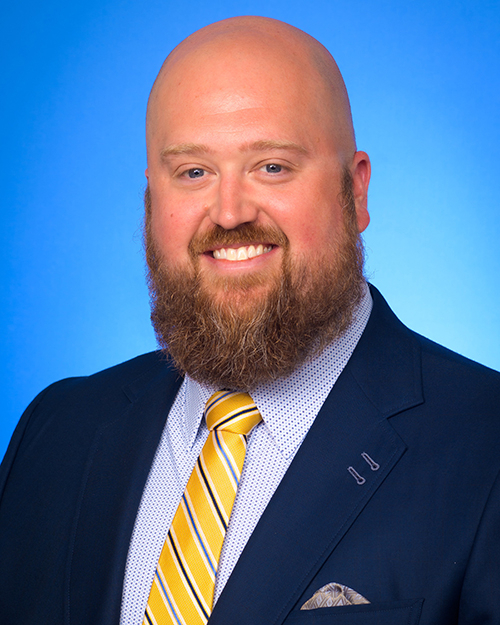

**Dr Burt**. In the real world, how deep a nodule can be detected by this? There must be some distance, I would imagine, from the visceral pleura where the accuracy may decline. Maybe not, but—

**Dr Baker**. Yeah. Within 2 cm we can reliably see it. Once you get beyond that, I mean, you can still see it, but there's a lot of factors that I've noticed that play into it. The size of the lesion, the deeper the lesion is, it's harder to see. But anything that's within 2 cm is pretty reliably visualized.

**Dr Burt**. And finally, in the real world, and I think this would be great for pure ground-glass opacities (GGOs), let's say pure adenocarcinoma in situ on pathology, I don't know if these premalignant lesions express the folate receptor. But I guess, how accurate is this for intraoperative nodule localization for those type of lesions?

**Dr Baker**. In terms of which cases that we primarily use that for now, ground glass opacity cases are 1 of the first where we really tried to clear that hemithorax. We really spent time scavenging when we were in there, because a lot of those occult lesions were in those patients. So I think, obviously, we don't know what that's going to play in the future for that patient, taking out all those lesions, but if we're in there, we have chosen to take out everything that lights up, and the majority of them are either malignant or premalignant.

**Dr Burt**. But the ground-glass opacities light up?

**Dr Baker**. Yeah.

**Dr Burt**. All right. Thank you so much.

**Unidentified Speaker 1**. Thank you very much.

## Conflict of Interest Statement

Dr Baker is a speaker for Stryker Medical. The other author reported no conflicts of interest.

The *Journal* policy requires editors and reviewers to disclose conflicts of interest and to decline handling or reviewing manuscripts for which they may have a conflict of interest. The editors and reviewers of this article have no conflicts of interest.

